# Interaction With Surface Spins as a Contribution to Nuclear Magnetic Relaxation of Liquids Adsorbed in Mesoporous Materials

**DOI:** 10.1002/mrc.70130

**Published:** 2026-07-01

**Authors:** Carlos Mattea, Hannah Leptin, Moritz Schmidt, Siegfried Stapf

**Affiliations:** ^1^ Dept. Technische Physik II/Polymerphysik Technische Universität Ilmenau Ilmenau Germany

**Keywords:** confinement, diffusion, NMR, phase separation, porous media, relaxation

## Abstract

NMR relaxation of liquids in the presence of solid interfaces, such as silica gel or porous glass, is affected by slowed‐down and anisotropic molecular reorientations compared to the bulk, but also by the presence of spins as potential relaxation partners on or close to the surface. The latter contribution is predominantly considered as unpaired electrons in metal centers or surface radicals, but dipolar interactions with spin‐bearing nuclei such as ^29^Si or ^1^H may also contribute to relaxation of adsorbed species. In this study, an attempt is made at identifying the most relevant relaxation contributions by either adding or removing relaxation sinks: While immobilized stable radicals (TEMPO) on the surface shorten relaxation times of adsorbed liquids, the replacement of ^1^H nuclei by ^2^H in hydroxyl groups is expected to remove the dominating part of nuclear dipolar spin–spin relaxation. In both cases, it is assumed that surface chemistry, which is the decisive parameter for spin relaxation mechanisms at interfaces, remains unchanged. Longitudinal and transverse relaxation, 2d T_1_‐T_2_ maps, and frequency‐dependent relaxation measurements of a number of homogeneous liquid phases as well as binary mixtures in the pore space of silica gel and porous glass are discussed in terms of the presence of radicals, whereas the replacement of OH by OD is observed to reduce relaxation rates in the systems under study.

## Graphical abstract

Liquids at silica interfaces experience demixing as a consequence of their respective polarity. The distribution of liquids and their primary relaxation contributions are studied by surface modification with OD and radical moieties, respectively.

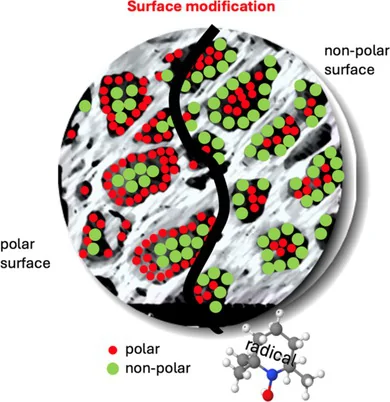

## 1 Introduction

Nuclear magnetic resonance (NMR) relaxation of liquids in macropores such as rocks and soils is strongly connected to wettability, a parameter that can be determined independently by, for instance, contact angle measurements in situations where a model substance of sufficiently flat surface can be obtained. A major tool in crude oil appraisal has for decades been the determination of relaxation times T_1_ and, more importantly, T_2_ which allows for a distinction of water and oil in a rock sample, even during downhole drilling. In general, water in a water‐wet environment will have a shorter T_2_ than oil, and vice versa. The molecular weight distribution in crude oil can require comparison with other parameters such as self‐diffusion in a 2D NMR experiment. Mixed‐wet conditions may not be resolved by this simplified approach, just as the influence of mineralogical composition of the rock such as the effect of metal oxides that can be diamagnetic or paramagnetic. The situation is somewhat different in artificial mesoporous materials where the concentration of paramagnetic centers can be so low that other mechanisms start to dominate NMR relaxation. Although differences in molecule‐surface interaction strength remain, the concept of wettability is often not considered. What is common to the interpretation of liquids in macro‐ and mesoporous materials is the assignment of an empirical specific surface relaxivity ρ_1_ or ρ_2_, a parameter that is suitable for obtaining information about surface‐to‐volume ratios and pore sizes but usually is not discussed in any detail. The fact that ρ_1_ and ρ_2_ are dependent on Larmor frequency, in the same way as all relaxation rates, is often ignored. However, a number of theories have been developed that attempt to explain that frequency dependence by means of modelling the underlying autocorrelation function of molecular orientation, G(t), and by making assumptions about the molecular motion in the immediate vicinity of the surface.

Both cases of either dominating paramagnetic relaxation, i.e., dipole–dipole coupling of molecular ^1^H nuclei with unpaired electrons at or immediately beneath the solid surface, or the absence of paramagnetic relaxation, have been presented, and lead to different T_1_ dispersions. While the former case is relevant for many natural rocks but also for cements and building materials [1, 2, 3], the latter can be found in synthetic glasses or clays, or on soft solids like hydrogels [4, 5, 6, 7, 8, 9, 10, 11]. In both approaches, however, the exact structure of the solid surface does not enter into the modelling: Paramagnetic relaxation in Korb's model [2] relies on the spatial distribution of the electron sites, whereas the opposite case is analyzed by Kimmich [9] assuming the geometrical structure of the surface via its curvature. Real cases may often be considered as an intermediate between both models, and the interaction with surface spins in principle needs to take into account all spin‐bearing nuclei on the surface. A similar approach as that suggested by Korb et al. may be used for this case, where the much smaller magnetic moment of the nuclear spins compared to that of electrons is partially compensated by the much higher nuclear spin density on the surface. Due to their large gyromagnetic ratio γ, ^1^H nuclei are expected to have the largest influence and occur mainly in OH groups when considering inorganic glasses.

Obtaining experimental evidence for the relative importance of these different contributions faces the problem of possibly also affecting the chemical properties, such as polarity or roughness, of the surface by the sample preparation. In this study, we suggest two attempts that need to consider this issue. First, liquids in commercial silica samples with and without TEMPO as radicals grafted to the surface are compared. In this case, identical surface chemistry and structure cannot be assumed a priori, but discussing qualitative trends and a comparison between different adsorbates appears feasible. Second, ^1^H nuclei in OH were replaced with ^2^H by exchange under mild conditions which guarantees that neither structure nor polarity of the surface are affected, unlike approaches where the OH group density is reduced by different types of treatment such as silylation. The results of relaxation properties will be discussed keeping the mentioned limitations in mind.

The properties of fluids in mesoporous media are known to be affected both by the presence of the solid interface and by the pore confinement itself; for instance, melting point depression, boiling point variations and changes in the overall rotational and translational dynamics are well described effects. Among experimental techniques that have been employed for studying confined liquids, NMR has demonstrated to be both versatile and sensitive to different aspects of molecular behavior, such as small‐scale (relaxation) and large‐scale dynamics (diffusion measurements), probes of the local environment (spectroscopy) and order (multi‐quantum experiments). While NMR relaxometry is sensitive to the immediate environment of the molecule and its reorientation with respect to the solid wall and its neighboring molecules, magnetic field‐gradient diffusion experiments measure average displacements on the order of 10^−7^ m and above. In combination, they have been employed to describe the dynamics of molecules over several orders of magnitude in time and length, in particular when low‐field or variable‐field NMR devices are used that extend the relaxation sensitivity towards slow molecular motions down to the kHz regime. However, while self‐diffusion properties predominantly sample geometrical effects by estimates of the apparent tortuosity, and pore size distribution information becomes available when the mean‐squared displacements are on the order of the pore sizes, relaxation requires the use of complex models in order to obtain actual information about the way molecules are affected by the solid interface. Although such model descriptions are continuously being improved for different types of solid materials, the general trends of confined fluids' relaxation are understood today: Strong interaction, such as for polar and/or protic liquids on polar surfaces, lead to much shortened relaxation times and a strong field‐ and frequency dependence T_1_(ω), whereas weakly interacting fluids do not show this effect, except when paramagnetic centers on or below the solid surface accelerate relaxation via nucleus‐electron spin interaction. This has also been consistently found by us both on macroscopic pores [12, 13] and in mesoporous environments [14, 15, 16]. The paramagnetic contribution can, however, be mostly excluded for many artificial mesoporous materials such as glass, silica gel, or ceramics.

To date, mixtures of liquids in mesoporous materials remain poorly studied, despite their importance in heterogeneous catalysis where either liquid–liquid reactions or the coexistence of a liquid reactant with its product may be encountered under certain reaction conditions. The accessibility of the pore wall, which contains the catalytically active metal sites, and the transport of reactants and products to these sites and away from the surface represent challenges which can be addressed by NMR relaxometry and diffusometry but apparently have not been studied in detail [17, 18, 19].

In this work, we build on recent findings of a possible demixing of certain combinations of liquids in mesoporous materials that are fully miscible in the bulk, e.g. acetone and cyclohexane, or tetrahydrofuran and cyclohexane [19, 20]. As the polar molecule acetone is preferentially adsorbed to the polar surface, it is suggested that relaxation of the polar and unpolar components in a binary mixture are affected differently by surface modifications, in particular by the presence of radicals.

## 2 Theoretical Background

Generally speaking, NMR relaxation is a consequence of spin energy transitions due to fluctuating magnetic fields in the vicinity of a nuclear spin, or electric field gradients in case the spin is of quadrupolar nature. Field fluctuations are predominantly brought about by motion inside the molecule or of the molecule itself with respect to its environment, in particular relative to the direction of the external magnetic field *B*_*0*_. It is thus often stated in a somewhat abbreviated, but essentially correct simplification that relaxation is described by the autocorrelation function of molecular orientation, *G(t)*. Under the assumption that molecules can be approximated as rigid bodies—internal dynamics of small molecules are sufficiently fast not to give a significant contribution to the field modulations—one can separate intra‐ and intermolecular contributions for dipolar nuclei where relaxation takes place by the dipolar coupling of two *I = 1/2* spins either within the same molecules, or in different molecules. For quadrupolar nuclei, the interaction with the electric field gradient usually dominates so that one can assume that relaxation is of purely intramolecular nature; comparing, for instance, relaxation in molecules containing either ^*1*^ *H* or ^*2*^ *H* thus serves as a simple means to estimate the relative strength of intra‐ and intermolecular relaxation in their different dependences on magnetic field strength.

Relaxation rates are proportional to transition probabilities between spin energy levels, the latter being expressed by the so‐called spectral density function *I(ω)* which itself is the Fourier transform of the mentioned autocorrelation function *G(t)*.

The total relaxation rate is a sum of all contributions, in particular intra‐ and intermolecular parts:

(1) $$R_{1,2}=T_{1,2}^{-1}=R_{\left(1,2\right)\text{inter}}+R_{\left(1,2\right)\text{intra}}$$

For both contributions, a specific dependence on the spectral density functions is observed. In particular, for dipolar coupling of like spins *I* and unlike spins *I,S* , the following expressions for *R*_*1*_ and *R*_*2*_ are found in the case of a fixed spin separation *r*:

(2) $$R_{1,\text{homo}}=C_{\mathrm{dip},\text{homo}}\;\left[I\left(\omega_{I}\right)+4\;I\left(2\omega_{I}\right)\right]$$

(3) $$R_{1,\text{hetero}}=C_{\mathrm{dip},\text{hetero}}\;\left[I\left(\omega_{I}-\omega_{S}\right)+3\;I\left(2\omega_{I}\right)+6\;I\left(\omega_{I}+\omega_{S}\right)\right]$$

(4) $$R_{2,\text{homo}}=C_{\mathrm{dip},\text{homo}}\;\left[\frac{3}{2}I\left(0\right)+\frac{5}{2}I\left(\omega_{I}\right)+I\left(2\omega_{I}\right)\right]$$

(5) $$\begin{array}{c} R_{2,\text{hetero}}=C_{\mathrm{dip},\text{hetero}}\\ \;\left[2I\left(0\right)+\frac{1}{2}I\left(\omega_{I}-\omega_{S}\right)+\frac{3}{2}\;I\left(2\omega_{I}\right)+3\;I\left(\omega_{S}\right)+3\;I\left(\omega_{I}+\omega_{S}\right)\right] \end{array}$$

with the prefactors $C_{\mathrm{dip},\text{homo}}={\left(\mu_{0}/4\pi \right)}^{2}\gamma^{4}\hbar^{2}I\left(I+1\right)/5r^{-6}$ for homonuclear dipolar relaxation and $C_{\mathrm{dip},\text{hetero}}={\left(\mu_{0}/4\pi \right)}^{2}\;\gamma_{I}^{2}\gamma_{S}^{2}\hbar^{2}S\left(S+1\right)/15r^{-6}$ for heteronuclear dipolar relaxation of the *I* spins by the *S* spins, where *I* and *S* are the spin quantum numbers, *r* is the internuclear distance, and *γ*_*I*_, *γ*_*S*_ are the gyromagnetic ratios of the spins involved, respectively.

Note that, apart from different combinations of the respective Larmor frequencies *ω*_*I*_, *ω*_*S*_, the relaxation rates are dominated by the prefactor *γ*_*I*_^*2*^ *γ*_*S*_^*2*^ which allows for an estimate of relaxation contributions due to dipolar couplings to different nuclei. Most importantly, γ_H_/γ_D_ ~ 6.5 so that a ^*1*^ *H* nucleus coupled to a ^*2*^ *H* nucleus has an about 40 times smaller relaxation rate compared to a ^*1*^ *H* ‐^*1*^ *H* pair, the relaxation rate contribution in a partially deuterated system is therefore often neglected.

The most relevant *S* spin contribution, if ^*1*^ *H* nuclei are observed, is unpaired electrons with their 658 times larger gyromagnetic ratio. The presence of unpaired electrons as part of metal impurities or radicals has been excluded on the basis of EPR (electron paramagnetic resonance) measurements carried out on bulk and silica samples in this study, in agreement with literature that confirms a negligible amount of paramagnetic impurities in many porous glass and silica materials, in particular in Vycor [1, 7]. The contributions of spin interactions by unlike spins are therefore negligible in this study except for the cases where radicals are deliberately introduced into the samples.

The spectral density functions for bulk liquids (Lorentzian for rotation and Hwang–Freed model for translation [21]) differ considerably from the available models for confined liquids. Focusing on the intermolecular contribution in Equation (1) allows for a possible distinction: As $R_{\left(1,2\right)\text{inter}}$ for dipolar relaxation is proportional to the total spin density, reducing the ^1^H density by partial deuteration of either the liquid or the surface will reduce $R_{\left(1,2\right)\text{inter}}$ and will thus lead to longer relaxation times (see below).

Broadly speaking, models for confined liquids fall into two classes, those where relaxation with unpaired electrons close to the solid surface dominate over other contributions [2, 3] and those with a negligible concentration of unpaired electrons, where relaxation takes place by molecular reorientation itself (dubbed reorientations mediated by translational displacements [RMTD]) [9]. The surface concentration of paramagnetic centers can be estimated experimentally from the volume‐averaged EPR line intensity under the assumption of a homogeneous distribution or is given by surface decoration as in this study. In agreement with earlier literature, Vycor porous glass is essentially “clean” [1, 7] so that this second model is more appropriate. RMTD, under these assumptions, does not take into account specific spin–spin interactions either with electrons or nuclei on the surface; it merely represents a limiting case that has been employed for a number of different porous materials, as mentioned earlier. The purpose of this study is not primarily to distinguish between the applicability of either model to the particular situation of silica and glass, but rather to estimate potential contributions by spin interactions with the surface and their influence on either approach.

## 3 Samples and Experiments

Liquids were obtained from the following sources and were used without further purification: acetone (99.8% purity), cyclohexane (99.9% purity), tetrahydrofuran (99.9% purity), and n‐decane (99% purity) by Carl Roth GmbH & Co KG, Karlsruhe, Germany; acetone‐d6 (99.5% deuterated) and cyclohexane‐d12 (99.5% deuterated) by Cambridge Isotopes Laboratories Inc.; tetrahydrofuran‐d8 (99.5% deuterated) by Deutero GmbH, Kastellaun, Germany. Bidistilled water was used and D_2_O (99.9% deuterated) was purchased from Deutero GmbH.

As porous materials, Vycor porous glass (Corning 7930) with a nominal pore diameter of 4 nm, fumed silica powder (product number S5130, Saint Louis, United States), SiliaFlash G60 (SiliCycle, ZeoChem, Quebec, Canada) with a nominal pore diameter of 6 nm, as well as SiliaCat with TEMPO (Sigma Aldrich, Saint Louis, United States) of nominal pore diameter of 5 nm were purchased. Vycor glass was obtained as cylindrical rods of approx. 3 mm diameter and was cut into pieces of 20–30 mm length. All silica materials were powders with particle diameters of 60–200 μm (SiliaFlash) and 125 μm (SiliaCat), or fumed powder with 7 nm size (silica gel Sigma‐Aldrich), respectively. The radical‐doped SiliaCat contained 0.8–0.85 mmol/g of 2,2,6,6‐tetramethylpiperidinyloxyl (TEMPO) grafted to the silica surface. All silica materials possessed specific surface areas between 370 and 500 m^2^/g, whereas Vycor is characterized by a specific surface of 250 m^2^/g.

For investigating the influence of radicals on liquid relaxation, the raw materials Vycor, SiliaFlash, and SiliaCat were used without modification but underwent a cleaning procedure to generate identical preparation properties. As a first step, silica was heated to 85 °C in deionized water on a hot plate while being stirred with a magnetic stirrer for 5 h to ensure complete dispersion and hydration. The silica was then centrifuged at 6000 rpm for 10 min to separate the silica from the water. After that, it was dried in an oven under vacuum at a temperature of 110 °C and a pressure of a few millibars for at least 16 h, and the resulting material was stored in a container sealed with parafilm in a desiccator under a fume hood until further use.

For the study involving surface hydroxyl variation, silica was treated as above and was subsequently modified in either of two ways. In order to exchange OH protons by deuterons and to generate OD surface hydroxyl groups, approximately 1 g of silica was added to 20 mL of deuterium oxide, and the heating/centrifugation cycle was carried out as described above; this exchange process was repeated once.

For silylation, chlorotrimethylsilane with a purity of 98%, purchased from ThermoFisher GmbH, was used. To prevent unwanted hydrolysis reactions and ensure the efficiency of the silylation process, 25 mL of n‐hexane was added to a reaction vessel as an anhydrous liquid together with 1 g of the previously dried silica. Maintaining an anhydrous environment is of importance because the presence of water can lead to unwanted side reactions that compromise the functionalization process. After heating the mixture to about 40 °C during constant stirring using a magnetic stirrer, 2 g of chlorotrimethylsilane was added. The reaction vessel was stirred under reflux in a fume hood at a temperature of 40 °C overnight to allow sufficient time for the reaction to proceed to completion. The functionalized silica was then collected by centrifugation at 6000 rpm for 10 min, followed by four runs of flushing with n‐hexane to remove unreacted reagents and by‐products. After each flushing cycle, centrifugation was carried out as described above to ensure efficient separation of the silica from the liquid phase [22].

The saturation of dry silica with the different liquids was achieved as follows: About 50–100 mg of the silica powder was transferred into an NMR tube of 5‐mm outer diameter, and the liquid, or liquid mixture, was added at an amount of about 70% by weight. After the liquid was added, the NMR tube was centrifuged to ensure thorough mixing. The powder was gently compressed using a glass rod for removing trapped air from the sample, and supernatant liquid was removed by a capillary. The NMR tube was subsequently flame‐sealed.

Vycor glass rods were first boiled for 60 min in a 30% H_2_O_2_ solution in a reflux condenser, followed by boiling in bidistilled water for another 60 min, and subsequently dried in vacuum at 110 °C for about 16 h to remove organic contaminants which are adsorbed from the atmosphere. This process was carried out twice and the cleaned samples were then placed in glass tubes filled with 400 μL liquid or liquid mixture for 24 h.

For OH/OD replacement of Vycor, samples were placed in a sealed container with approximately 15 mL of deuterium oxide per 1 g of Vycor and left for 5 days to ensure complete isotopic exchange before proceeding with the drying process. The silylation of Vycor was carried out in the same way as for silica, using 2 g of chlorotrimethylsilane and 25 mL of n‐hexane per 1 g of Vycor. In the case of Vycor, the flushing process was conducted over a period of 2 days in a container filled with n‐hexane, with constant stirring using a magnetic stirrer, and the n‐hexane was replaced four times during this period. This extended cleaning process ensures that no unreacted reagents or by‐products remain on the surface, since centrifugation is not applicable to Vycor. After drying the surface‐modified Vycor rods in an oven under vacuum under the described conditions for 16 h and letting them cool to 40 °C under reduced pressure, the samples were prepared immediately.

Prior to measurements, glass rods were taken out of these tubes and wiped superficially to remove bulk liquid; they were then placed in either 5 mm OD (one piece) or 10 mm OD (three pieces) NMR tubes. The empty volume above the Vycor rods was filled by a solid glass rod in order to reduce the volume for a possible vapor phase; tubes were then flame‐sealed.

Measurements of signal intensity via ^1^H spectra, and of ^1^H relaxation times (T_1_, T_2_) were carried out at a SpinSolve 1.0 T spectrometer (Magritek, Aachen, Germany) at a resonance frequency of 42.6 MHz set to T = 300 K employing standard 1d and 2d experiments and using 200 μs pulse separation in the 1d‐Carr–Purcell–Meiboom–Gill (CPMG) sequence. In the 2d T1‐T2 experiment, a combination of inversion‐recovery pulse sequence with a CPMG pulse sequence was used in the following way: the free induction decay is acquired in the interval between the 90° pulse and the first 180° pulse, with a dwell time of 1 μs, and combined with the CPMG echo decay signal, to encode the T_2_ relaxation. The analysis follows a procedure described earlier [23, 24, 25]. Data were analyzed by multiexponential functions and by the so‐called ILT algorithm implemented in Matlab that in essence carries out a numerical solution of Fredholm integral equations of the first kind to generate a probable distribution function. In the following discussion of results, this will be abbreviated by the expression “ILT transform.”

Relaxation dispersion measurements were carried out at room temperature (293 K) on a Stelar Fast Field Cycling (FFC) SpinMaster 2000 relaxometer (Stelar, Mede, Italy) at magnetic field strengths between 0.02 mT and 0.7 T with the detection field set to 0.26 T. Signal acquisition was achieved by a CPMG pulse sequence, adding up echo intensities to improve SNR, and using typically four accumulations for each time value. All T_1_ relaxation curves were found to follow a monoexponential function.

## 4 Results

### 4.1 Influence of Surface Polarity and Surface Protons on the Relaxation

The concept of replacing surface OH groups by OD is possibly the simplest test of assessing the importance of a coupling of the adsorbed liquid with the solid surface. This includes chemical bonds, hydrogen bonds, and dipole–dipole relaxation contributions, where the hydroxyl hydrogen is expected to be the main contributor due to its large gyromagnetic ratio and the absence of any significant amount of further ^1^H nuclei near the surface. If any actual bonds were present, replacement of ^1^H by ^2^H does not significantly alter their strength, although the hydrogen bond mechanism will affect the hydrogen distribution in protic liquids such as water. A dipolar relaxation contribution to both *R*_*1*_ and *R*_*2*_, on the other hand, will be scaled down by a factor on the order of (γ_D_/γ_H_)^2^ or about 1/40, not considering the different prefactors entering the description for homo‐ and heteronuclear relaxation. This makes ^1^H‐^2^H dipolar relaxation essentially negligible for the majority of situations, so that a pronounced reduction of both relaxation rates is expected to be observable if this mechanism were of any importance for the total relaxation rate of adsorbate's nuclei. Silylation, on the other hand, is an established modification process but changes both the ^1^H density and the polarity of the surface; therefore, possibly affecting the entire reorientation dynamics of the adsorbate. The results on silylated surfaces are thus presented only in comparison with the goal of identifying trends for different adsorbed species.

Prior to the relaxation experiments, the amount of OH protons exchanged during the described procedure was estimated by subjecting the treated silica and Vycor samples to a surplus amount of D_2_O; for the parameters chosen in the experiment, only liquid‐like ^1^H signal could be detected. Subtracting a baseline for residual ^1^H nuclei in D_2_O, the evolution of the signal during 5 h is presented in Figure 1. Estimating the surface OH density to be 5 nm^−2^ [26], this corresponds to a ratio of 8 mmol D_2_O in liquid vs. 0.2 mmol OH/OD on silica, and 10 mmol D_2_O in liquid vs. 0.5 mmol OH/OD in Vycor, respectively. The obtained signal stemming predominantly from ^1^H nuclei in HDO molecules is therefore, to a good approximation, proportional to the amount of exchangeable OH on the surface. It can thus be concluded that the concentration of ^1^H nuclei in surface‐bound hydroxyl groups is reduced to 43% in Vycor and to 52% in silica, respectively, by the exchange treatment. The temporal evolution of the signal rise suggests different exchange dynamics due to the geometry of the porous systems, an observation that need not be discussed at this stage. Relevant is only the fact that after 300 min, OH/OD exchange is completed and an equilibrium distribution is achieved.

**FIGURE 1:**
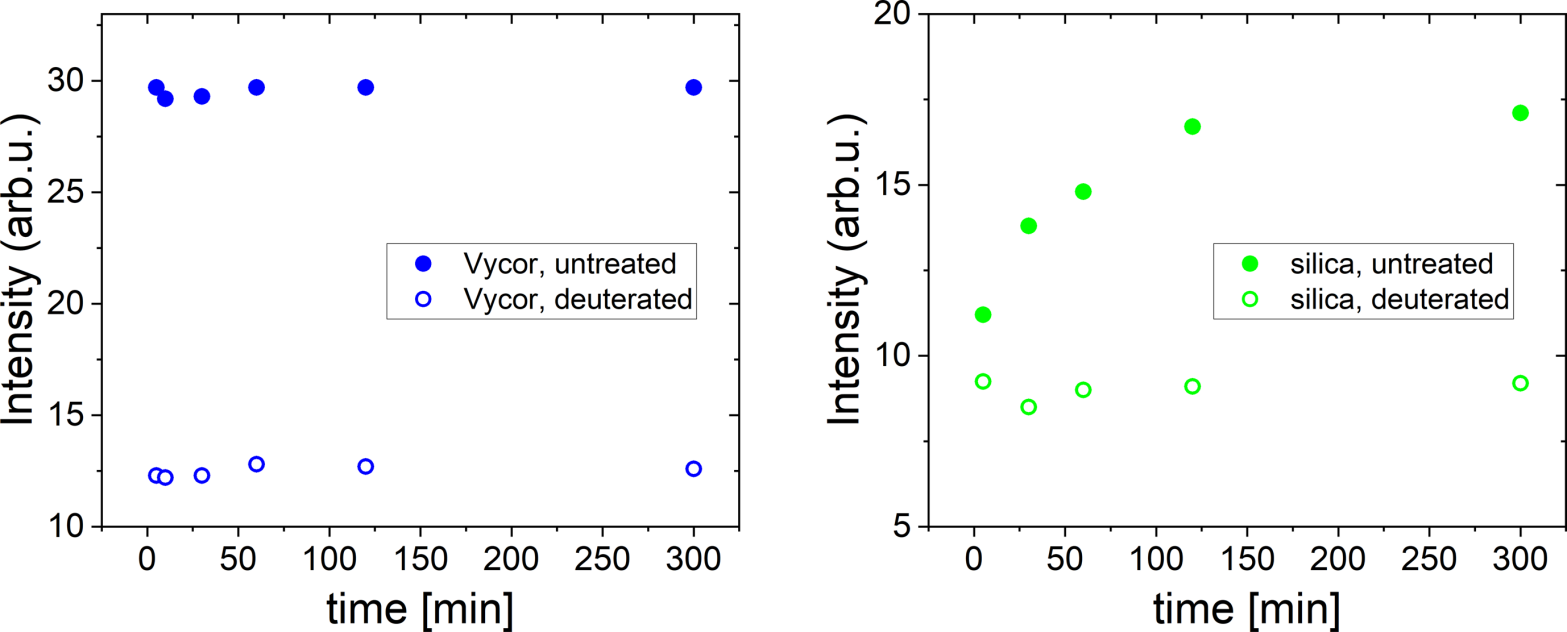
Liquid contribution to the ^1^H signal of D_2_O filling porous media with and without prior H/D exchange treatment as a function of time—left: Vycor; right: silica fumed particles. Signal is proportional to ^1^H nuclei exchanged from the surface.

Determining the signal intensity from the ^1^H spectra resulted in similar values of OH/OD exchange in the hydroxyl groups, and a reduction of the OH concentration to 40%–50% was obtained by silylation in Vycor, although these values are not strictly quantitative since small changes of *T*_*2*_ may affect the signal intensity under the experimental conditions used. Considering this partial removal of ^1^H in the surface hydroxyl groups, it can be expected that a possible dipolar relaxation contribution will be scaled down by a factor of about 1.9–2.3 for these samples.

Whereas the adsorbed amount of liquid obtained from spectra remained—within 20% error—the same for n‐decane, cyclohexane, tetrahydrofuran and acetone, even this partial silylation achieved rendered the surface so hydrophobic that only about 20% (silica) or 1% (Vycor) of the equilibrium amount of water was adsorbed into the samples, so that a direct comparison of the water relaxation data in the silylated media does not appear meaningful.

Relaxation times could be characterized sufficiently well by monoexponential decays with the exception of water ^1^H where two components were frequently identified. Figure 2 compares the longitudinal relaxation times for different liquids. At the chosen Larmor resonance frequency of 43 MHz, a slight but consistent increase of *T*_*1*_ is found for all liquids in the H/D exchanged samples, with a range of 5%–10% in Vycor and 10%–45% in silica, which also includes the weighted average of the two fitted *T*_*1*_ components of water in Vycor. Silylation did not result in an identifiable trend, in particular with respect to molecular polarity. As mentioned above, silylation led to a much lower water filling so that relaxation times cannot be compared directly for water.

**FIGURE 2:**
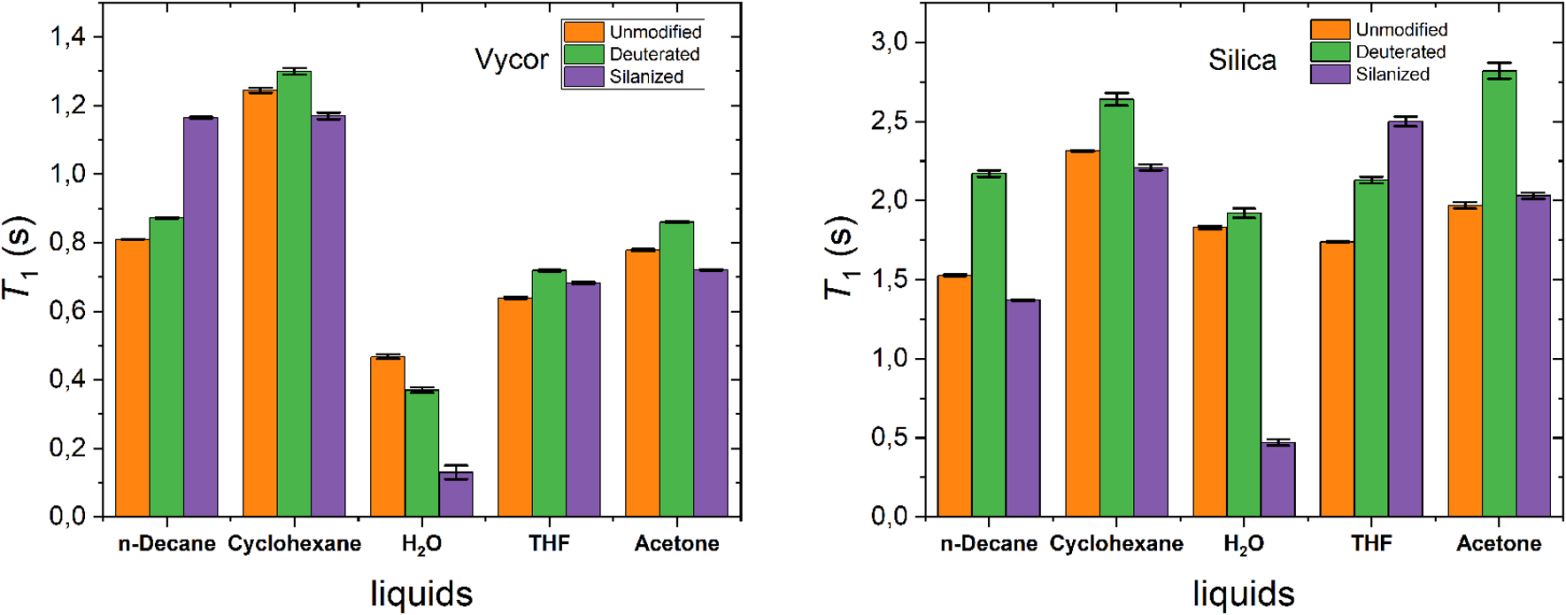
Longitudinal ^1^H relaxation times *T*_*1*_ at 1.0 T and 300 K for liquids in differently treated porous media.

By comparison, the effects on the transverse relaxation time, *T*_*2*_, are somewhat more pronounced, as shown in Figure 3. Whereas OH/OD exchange consistently leads to a 10%–30% reduction of *T*_*2*_ in Vycor, the opposite tendency is found in fumed silica where *T*_*2*_ become longer by factors between 1.3 and 2 except for water where a longer component in the untreated sample seems to disappear. Under the assumption that the samples' polarity remains unchanged, OH/OD exchange should have either no effect, if direct relaxation with hydroxyl protons is negligible, or should lead to a longer T_2_ in the opposite case. Under the assumption of slow (i.e., on the order of the reciprocal Larmor frequency, i.e., *τ* > 10^−8^ s) molecular reorientation processes becoming relevant, the variation of *T*_*2*_ is expected to be larger than in *T*_*1*_; in this respect, the results in silica are in agreement with a non‐vanishing contribution of the dipolar relaxation with surface hydroxyl protons to the total relaxation rate. The trend in Vycor, however, cannot be explained by this assumption (see Discussion).

**FIGURE 3:**
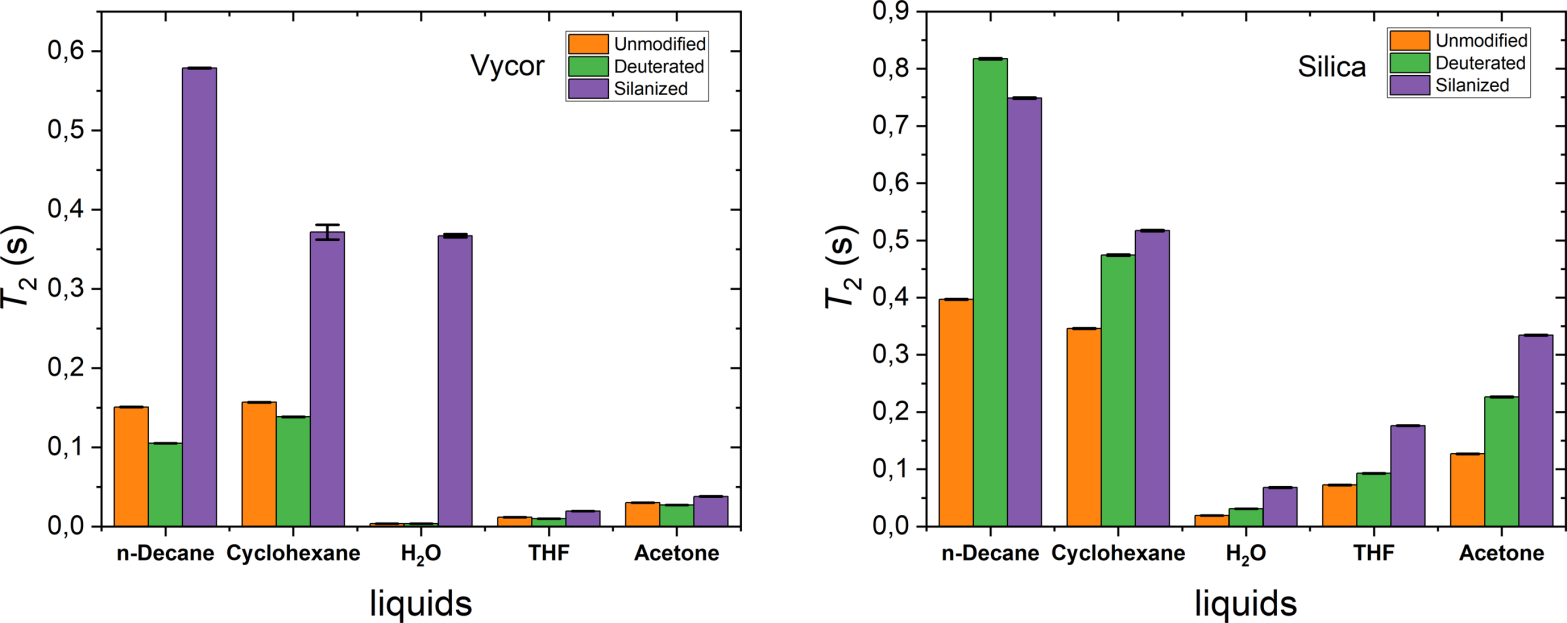
Transverse ^1^H relaxation times T_2_ at 1.0 T and 300 K for liquids in differently treated porous media.

Silylation, on the other hand, reduces the surface polarity while, at the same time, enhancing the surface ^1^H density by means of semi‐mobile CH_3_ groups. In many studies, it was observed that polarity is the decisive factor for relaxation properties [4, 27]; this also seems to be the case in the present situation where silylated samples lead to an increase of T_2_ by factors between 1.5 and 4 in Vycor and 1.5–2.6 in silica. This trend, however, is found for polar and non‐polar liquids alike.

Relaxation affected by slow motion, in the context mentioned above, is equivalent to a significant frequency dependence of the spectral density function, *I*(ω). Equations (2) and (4) suggest that *T*_*2*_ can be considered to be equivalent, apart from a constant pre‐factor, to *T*_*1*_ in the limit of zero frequency. Therefore, the ratio *T*_*1*_ */T*_*2*_ at a given frequency serves as a rough estimate of the strength of this frequency dependence. As becomes obvious from the separate trends of *T*_*1*_ and *T*_*2*_ discussed above, there is also no significant trend in their respective ratios which vary by no more than a factor of 1.5 for OH/OD exchanged samples—see Table 1. For silylated materials, the effect is somewhat more pronounced, with a generally weaker frequency dependence in all treated samples.

**TABLE 1:** Relaxation times ratios between untreated (“u”) and silylated (“s”) samples. Values for water are not directly comparable since they represent unsaturated samples due to strong hydrophobicity (1% filling fraction for silylated Vycor and 20% filing fraction for silylated silica).

Sample	Unmodified vs. silylated Vycor			Unmodified vs. silylated silica		
	T_1,u_/T_1,s_ T_2,u_/T_2,s_ (T_1,u_/T_2,u_)/(T_1,s_/T_2,s_)			T_1,u_/T_1,s_ T_2,u_/T_2,s_ (T_1,u_/T_2,u_)/(T_1,s_/T_2,s_)		
n‐Decane	0.70	0.26	2.7	1.1	0.53	2.1
Cyclohexane	1.1	0.42	2.5	1.0	0.67	1.6
Water	*2.5*	*0.01*	*260*	*2.4*	*0.27*	*8.9*
THF	0.94	0.60	1.6	0.7	0.41	1.7
Acetone	1.1	0.80	1.4	0.97	0.38	2.6

In order to identify a possible variation of the spectral density *I(ω)*, and therefore of the reorientation dynamics relevant for relaxation, field‐cycling relaxation measurements were carried out for selected systems. The difference between samples containing OH and OD was generally found to be rather small, and *T*_*1*_ *(ω)* dispersions followed parallel shifted functions. Figure 4 shows the comparison of tetrahydrofuran relaxation in untreated and silylated silica. While treatment leads to an about twice longer relaxation time, suggesting weaker interaction with the less polar surface, the shape of the dispersion remains the same, i.e., the mechanism of relaxation—possibly the RMTD process judging from molecular properties and the amount of dispersion—is not affected. The measured *T*_*1*_ is in agreement with the dominating component in the 2d measurement carried out at 1.0 T (see below) and the lack of a second component, as in all other variable‐field measurements, is due to the lower signal‐to‐noise ratio of this instrument.

**FIGURE 4:**
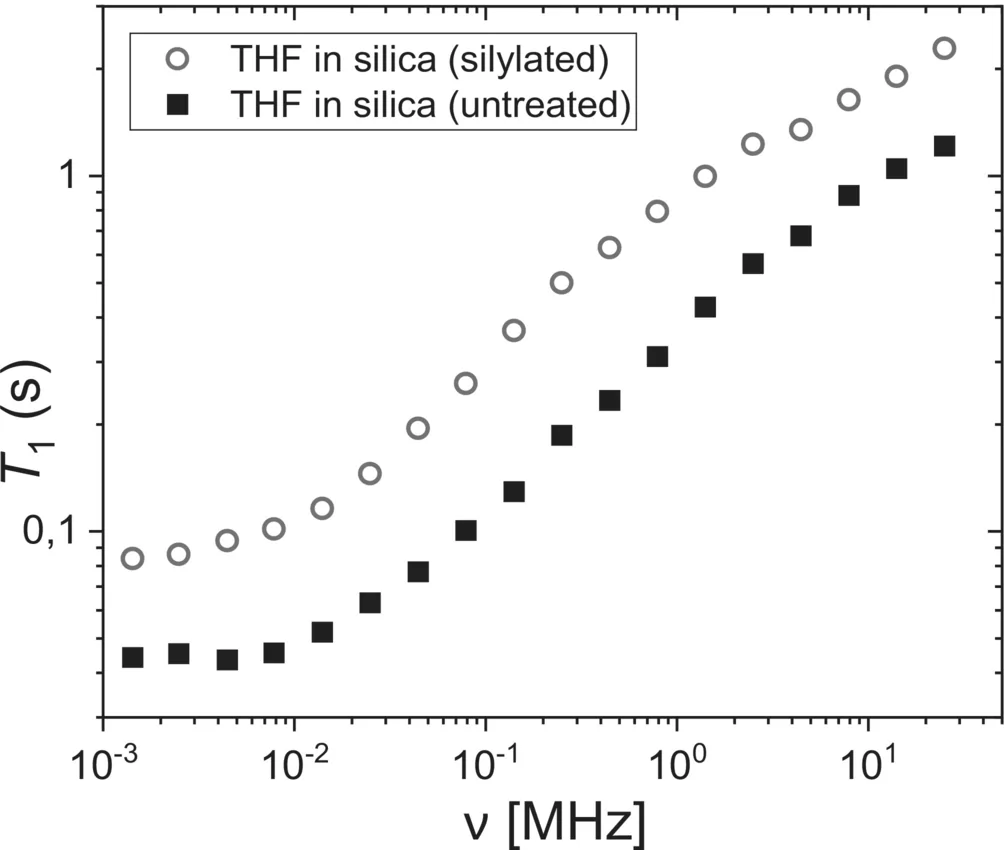
Frequency dependence of ^1^H *T*_*1*_ relaxation times for tetrahydrofuran in native and silylated silica.

Only a weak variation of T_1_(ω) was found for n‐decane in silica (see Figure 5) where the silylated sample leads to a less pronounced dispersion compared to the deuterated and also the untreated samples. Note, however, that unpolar liquids such as n‐decane are not expected to follow RMTD due to their low degree of preferential orientation and that the T_1_ dispersion generally remains rather weakly pronounced (note, however, much stronger dispersion that has been found in silica of larger specific surface and possibly more regular pore structures [16].

**FIGURE 5:**
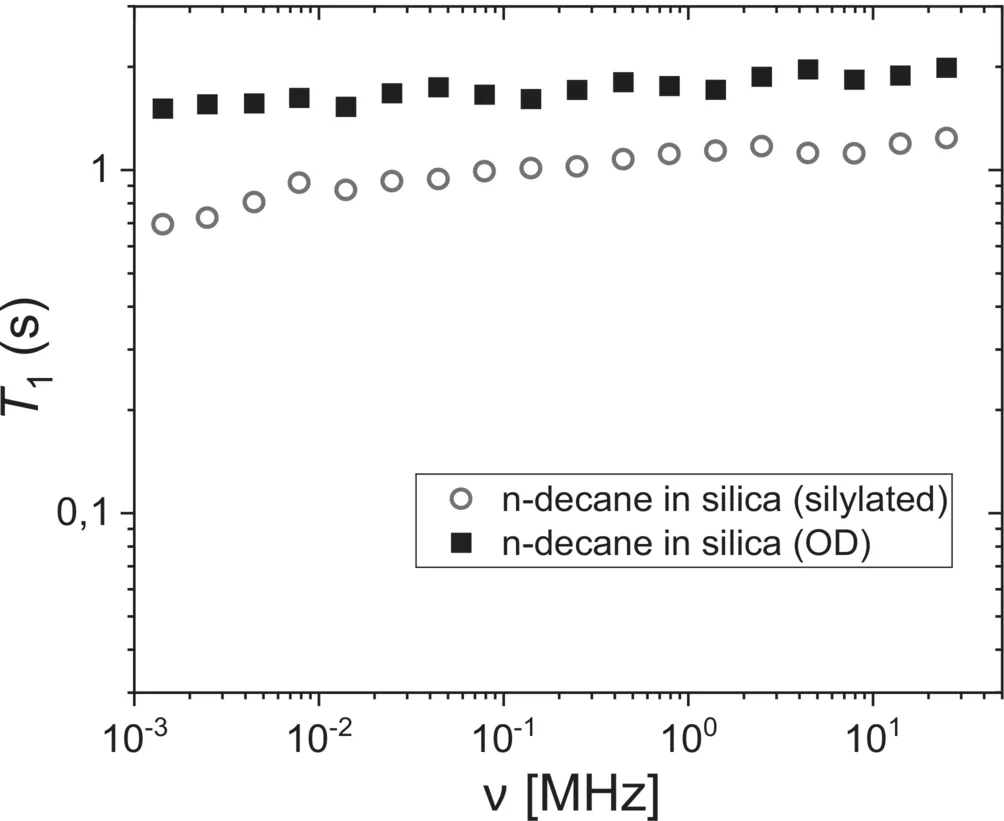
Frequency dependence of ^1^H *T*_*1*_ relaxation times for n‐decane in OD exchanged and silylated silica.

### 4.2 Influence of Surface Radicals on the Relaxation

Dipolar interaction with unpaired electrons constitutes the dominating relaxation mechanism for ^1^H nuclei if the electrons' concentration is sufficiently high, and they can be physically approached by the adsorbates' nuclei; at the very high concentration of TEMPO present in the studied samples, with an average separation of about 1 nm between tethered TEMPO radicals, this is expected to be true. At the same time, as in the previous case of silylation, the presence of radicals changes the surface chemistry and therefore potentially the reorientation dynamics of the adsorbate. The goal of this part of the study is twofold: firstly to quantify relaxation enhancement in single‐component liquids and identify heterogeneities of the molecular environment, secondly to search for signs of differential relaxation behavior in two‐phase liquids undergoing phase separation.

In the presence of radicals, spectral lines at B = 1.0 T were found to be too broad to allow for a safe distinction between different species; in the mixtures, one component was therefore introduced as a deuterated substance. Either acetone or tetrahydrofurane as polar liquids were mixed with unpolar cyclohexane in volume ratios of 10:90, 50:50, and 90:10, resulting in 12 mixture pairs depending on which component was deuterated. Actual liquid concentrations were estimated from the ^1^H signal intensities and were within a tolerance of under 5% relative to the total liquid amount, the deviation possibly arising from partial evaporation during the preparation process.

All measurements were carried out in a 2d mode T_1_‐T_2_ but were also verified by one‐dimensional experiments (inversion recovery and FID/CPMG, respectively); results of the 1d bi‐ or triexponential fits are, in all cases, in very good agreement with the local maxima of the corresponding 2d experiments. Figure 6 presents two examples for comparisons between the two types of silica: n‐decane and acetone as representatives of unpolar and polar liquids. The results for cyclohexane, tetrahydrofuran, and water showed the same qualitative features despite different values for T_1_ and T_2_. In all untreated silica samples (left‐hand side), decays are characterized by a dominating component on the order of 1 s in T_1_ and several 100 ms in T_2_, with shorter T_2_ being observed for polar species in agreement with earlier studies of silica materials [14, 15, 16, 28, 29]. A second component is found that is about one order of magnitude shorter in both relaxation times and of between 10% and 15% relative intensity. For both components, the ratio T_1_/T_2_ is between 1.5 and 4, with the larger values found for polar substances. Note that intensities at the border of the chosen relaxation range are common artefacts of the inversion algorithm, a supposition that was proven by varying the range of values of T_1_, T_2_ deliberately; this refers to apparently very long T_1_ and very short T_1_ values in Figures 6, 7, and 8.

**FIGURE 6:**
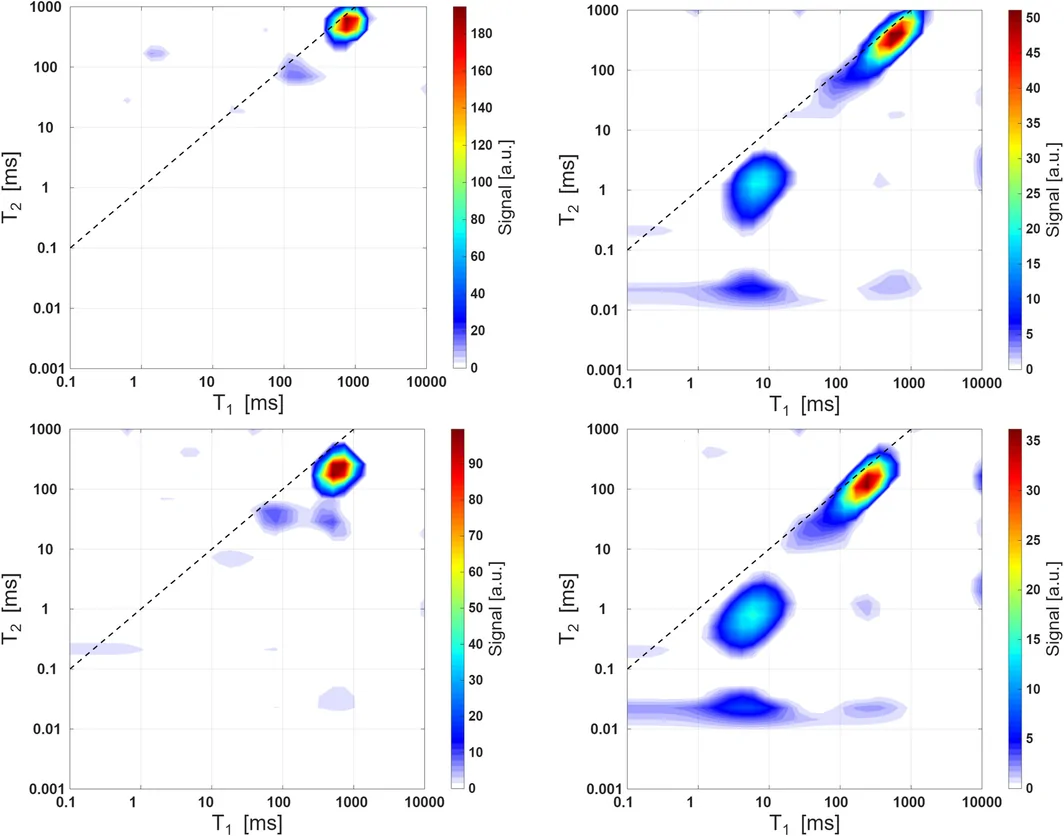
^1^H relaxation time distributions P(T_1_,T_2_) at a magnetic field strength of 1.0 T as obtained by Inverse Laplace Transform (ILT). Top: n‐decane; bottom: acetone. Left: silica without radicals; right: silica with TEMPO radicals. For comparison, the equality T_1_ = T_2_ is indicated by a dashed line.

**FIGURE 7:**
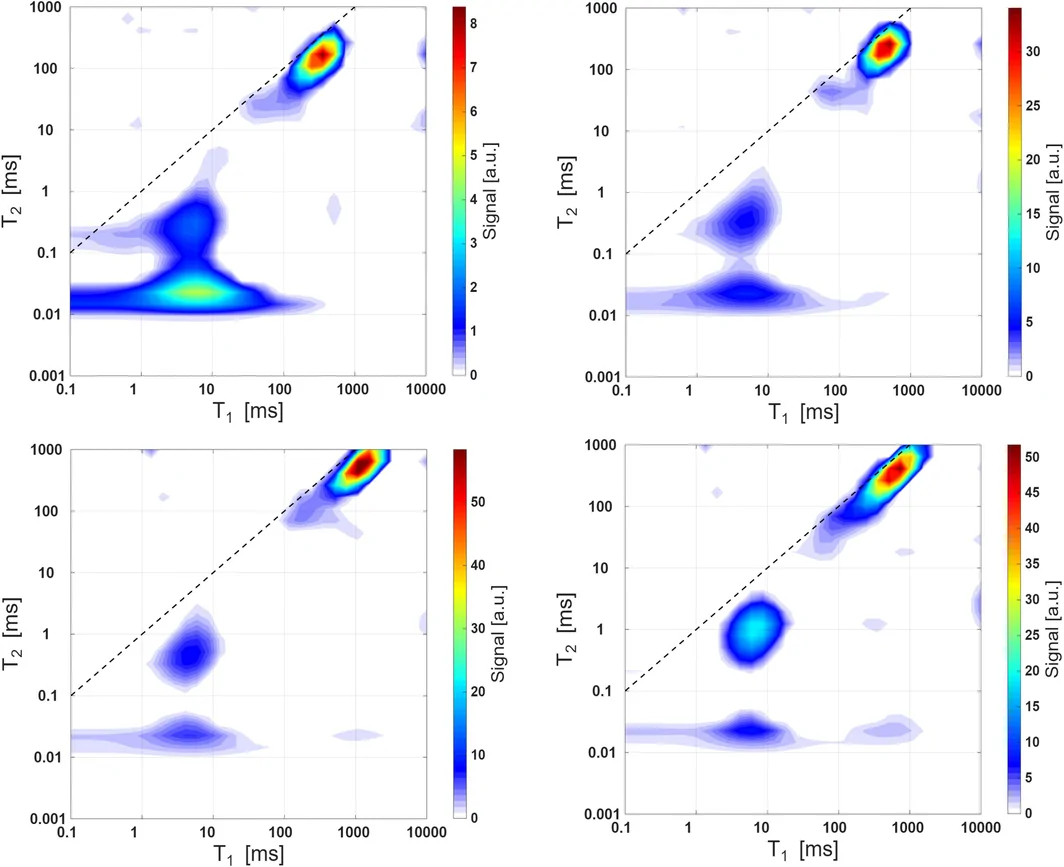
^1^H relaxation time distributions P(T_1_,T_2_) at a magnetic field strength of 1.0 T as obtained by inverse Laplace transform (ILT) for a mixture of cyclohexane and deuterated acetone in silica containing TEMPO at different volume percentages (top left, top right, bottom left, bottom right): 10, 50, 90, 100%. For comparison, the equality T_1_ = T_2_ is indicated by a dashed line.

**FIGURE 8:**
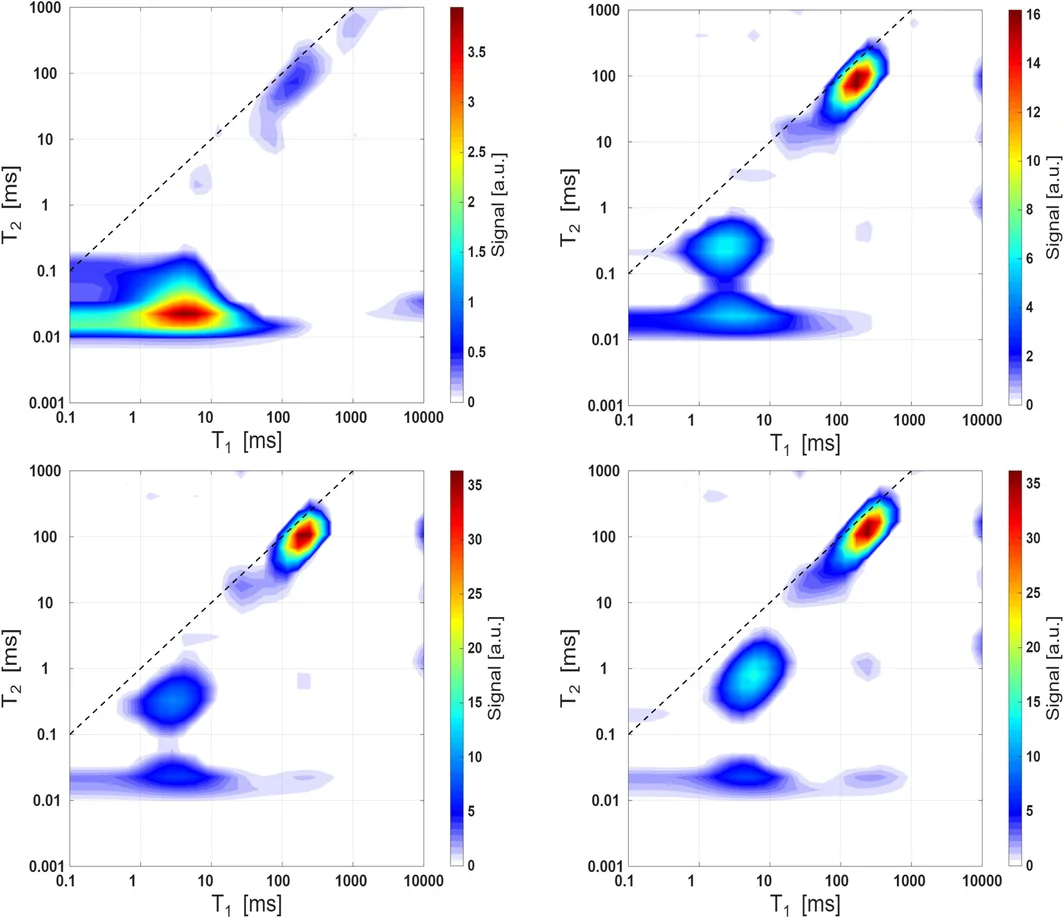
^1^H relaxation time distributions P(T_1_,T_2_) at a magnetic field strength of 1.0 T as obtained by inverse Laplace transform (ILT) for a mixture of acetone and deuterated cyclohexane in silica containing TEMPO at different volume percentages (top left, top right, bottom left, bottom right): 10, 50, 90, 100%. For comparison, the equality T_1_ = T_2_ is indicated by a dashed line.

In silica doped with TEMPO (right‐hand side of Figure 6), a shift towards shorter relaxation times is observed, but the effect is more subtle than possibly expected, perhaps a consequence of the inherent heterogeneity of liquid distribution in silica. The main peak is shifted towards shorter T_1_ and T_2_, but at the same time, it is becoming wider and extends towards the region where the short component is identified in the untreated silica samples. In the one‐dimensional experiments, a third component could be fitted to the data which remains inside the continuous area of the 2d‐fit and amounts to about 25%–40% relative weight, compared to 45%–60% of the component describing the longer times. In addition, a peak of about 10%–15% relative weight arises at much shorter T_1_ and T_2_. For all five liquids studied, this peak shows T_1_ between 4 and 8 ms and T_2_ between 0.6 and 1.6 ms, respectively, and the ratio T_1_/T_2_ is somewhat enhanced at 5–10. While there is no definite proof that the short‐time peak in the samples containing TEMPO has the same origin as the short‐time peak in the untreated silica, it seems reasonable to assume that it stems from regions in the samples where liquid is in direct contact with the surface, while the dominating peak suggests bulk‐like behavior which is affected only in a limited fashion by the presence of surface or TEMPO.

Note that the occurrence of an even shorter component about 20 μs in T_2_ is consistently above noise level in most experiments; it can be attributed to the surface‐bound OH groups and has been found in reference measurements of dry samples, but it cannot be analyzed quantitatively since it undergoes severe attenuation due to the instrumental deadtime.

Binary mixtures were studied in order to assess the feasibility of identifying possible preferential adsorption or microphase separation: If both liquids remain in a homogeneous mixture, they should be affected by the presence of silica surface and/or TEMPO in a similar fashion as the pure substances, but in case of phase separation, differences should become observable.

The 2d results for the pair acetone‐cyclohexane are shown in Figures 7 and 8, respectively. At lower concentrations of cyclohexane in acetone‐d6 (Figure 7), the peaks at long relaxation times tend to shift towards shorter values, and the short relaxation component shows a similar trend. For the polar liquid, acetone in cyclohexane‐d12 (Figure 8), the tendency appears to be similar, although the peak of the shortest *T*_*2*_ is beginning to merge with the background OH signal at lower acetone concentrations so that proper analysis is not possible any longer. The behavior of the pair THF/cyclohexane is qualitatively the same (data not shown). While the variations of the long relaxation component do not exceed a factor of 2, the range is somewhat larger for the short relaxation component. Figure 9 summarizes and compares the dependence of the latter for all studied mixtures.

**FIGURE 9:**
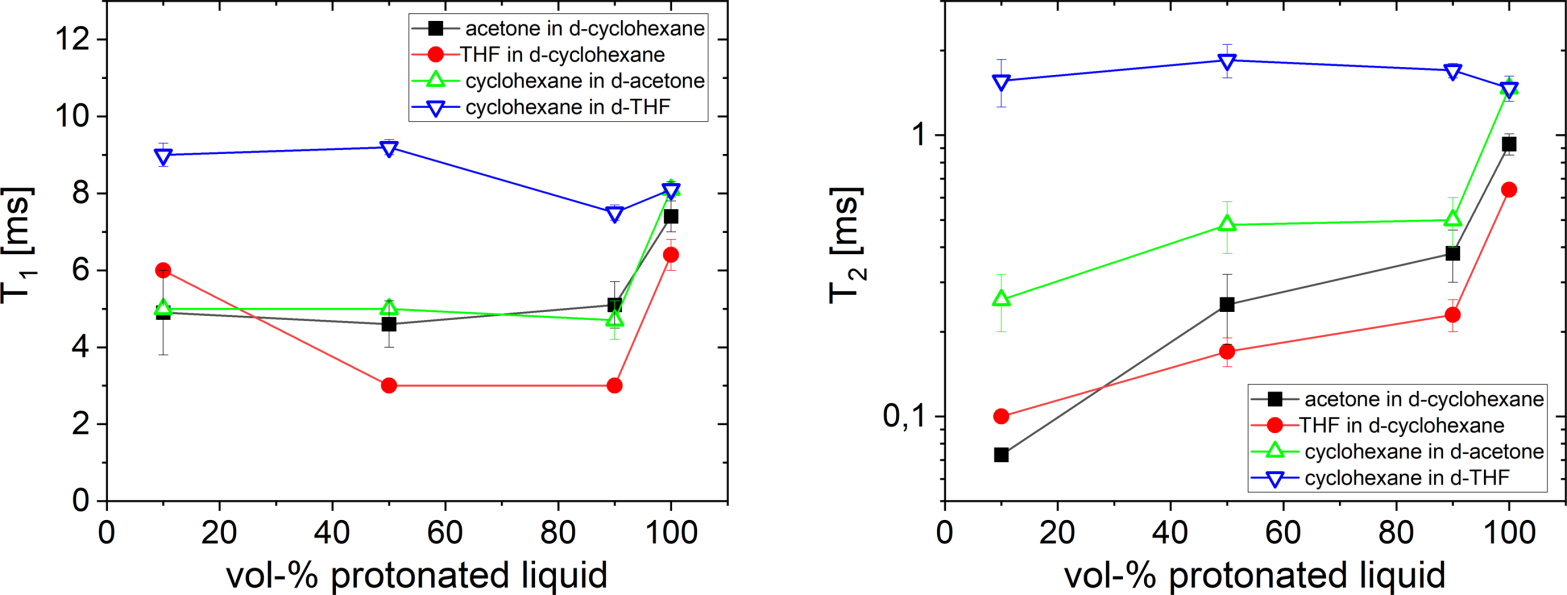
^1^H relaxation times (*T*_*1*_ *, T*_*2*_) of the shortest component at a magnetic field strength of 1.0 T for liquid mixtures in silica with TEMPO for one component mixed with the second, deuterated component at different volume percentages: 10, 50, 90, 100%.

The results of these measurements appear not entirely consistent, although the possibility of errors introduced by the fitting process needs to be taken into consideration. At a reduced amount of the proton‐containing liquid, *T*_*1*_ remains rather constant for cyclohexane but increases for acetone and THF; this appears to be in disagreement with the notion of polar molecules preferentially adsorbing to the surface where they are in contact with the radicals. In *T*_*2*_, however, the trend is opposite with *T*_*2*_ of the polar species being reduced about tenfold compared to the samples filled with the pure liquid. A predicted “shielding” effect, avoiding contact of the unpolar cyclohexane with the surface, is not observed, in seeming contradiction with results obtained in glass and alumina, [17, 19, 20], but see Discussion.

## 5 Discussion and Conclusions

NMR relaxation of ^1^H nuclei in liquids adsorbed on silica surfaces is primarily governed by polarity, but the structure of the porous material is also crucial for the interpretation of the relaxation process. While porous glass and fumed silica with pores or spherical particles on the nm‐scale can result in a narrow distribution of relaxation times due to fast exchange between surface sites and bulk‐like liquid [30, 31], the heterogeneity in silica beads allows for the coexistence of at least two environments inside the pores and between the macroscopic particles. In this study, bead sizes for SiliaFlash and SiliaCat are between 60 and 200 μm, on a similar scale as the diffusion length of liquids during the duration of the experiment which is approximated by (6DT_1_)^0.5^ or on the order of 100 μm, so that complete fast exchange is no longer expected, which explains the occurrence of two or three relaxation components in all cases. While no definitive assignment is attempted and we particularly study the trend during variation of sample conditions, it is likely that the components correspond to bulk‐like and intraporous liquid and OH/silyl protons, respectively.

For porous glass and fumed silica, surface treatment was found to affect relaxation properties by factors of up to 4, with silylation providing a stronger effect that can be attributed to it changing the polarity of the surface as a whole. In this study, both silylation and deuteration of surface OH groups were found to be incomplete and reaching a replacement amount of about 50% for both modifications. Still, a trend towards the expected behavior of relaxation was found, with relaxation times shorter and relaxation dispersion *T*_*1*_ *(ω)* more pronounced for unpolar liquids, and vice versa for polar liquids. This is in agreement with an early study of alumina pores undergoing coking with organic material, where the effect on *T*_*1*_ *(ω)* occurred in a strongly nonlinear dependence of surface coverage, beginning at low nominal coverage [27].

On the other hand, it could be confirmed in this study that replacement of OH in surface hydroxyls by OD does have a much weaker effect on ^1^H relaxation itself, not being proportional to the reduction of the surface ^1^H density to one half, since surface polarity was not changed and the contribution of dipolar relaxation of adsorbate protons with OH protons is apparently rather small. At this stage, it cannot be concluded with certainty that the, on average, small reduction of relaxation times in OD‐exchanged Vycor glass is systematic or the consequence of some other unknown surface alteration that was brought about despite the rather mild treatment of samples during exchange. Despite the time‐independent signal intensity shown in Figure 1, which suggests that an equilibrium OD concentration was reached, it remains possible that the degree of deuteration in these macroscopic samples is heterogeneous; further experiments with a more thorough sample modification are currently in progress for solving this issue.

More generally, the findings of only small variations in relaxivity upon OH/OD exchange support the concept of the RMTD mechanism as the significant relaxation contribution in the low magnetic field range under study. This concept had been developed for “clean,” i.e., metal‐free, surfaces of porous glass and silica and suggests that reorientations of the molecules, mediated by the surface structure which generates anomalous diffusion [32], are solely responsible for relaxation dispersion in the absence of any specific dipolar interactions with external spins. Nevertheless, as mentioned above, future studies will be required in which a more thorough OH replacement preparation of the porous material must be attempted, and the contribution of OH‐H dipolar interactions be quantified for aprotic as well as protic liquids with the support of MD simulations. The modulation, and effective averaging, of O^1^H relaxation in water and alcohols by means of exchange with surface OH groups has already been described in similar materials [33].

In a binary mixture, under the assumption of complete phase separation due to preferential adsorption of the polar species, the thickness of the adsorbed layer is estimated between about 0.1 and 2 nm. Since the TEMPO radical is tethered to the surface groups by a linker, the distance of the unpaired electron itself can be estimated to be between 0.8 and 1.4 nm from the surface, the latter value corresponding to an extended position pointing away from the surface. It can therefore not be expected that the polar liquid layer is sufficiently thick to fully hide the radical from the cyclohexane molecules; on the other hand, polar molecules might be removed away from the unpaired electron if they are too close to the surface, suggesting that small concentrations actually lead to less contact with the radical and therefore *longer* relaxation times as a consequence of the effectively less polar surface, in agreement with the *T*_*1*_ results. For *T*_*2*_, the nature of reorientation processes is relevant, and it cannot be assumed a priori that the tethered radicals do not affect the dynamics of adsorbed molecules. On the other hand, the opposite case of a homogeneous mixture can indeed be excluded: under these conditions, intermolecular relaxation will depend on the concentration of the deuterated species, but this also means that in the case of dominating intramolecular processes as suggested for silica and other materials with large specific surfaces, essentially no concentration dependence at all should be expected since the orientation of a polar molecule relative to the surface does not depend on its immediate liquid environment. This statement is actually valid irrespective of the type of model used, since intermolecular dipolar relaxation does not enter into either the RMTD or the Korb model. The observed relaxation properties for binary mixtures thus strongly suggest demixing of the liquids on the timescale of the relaxation experiment, as has been confirmed by MD simulations and experiments of acetone/cyclohexane mixtures in porous glass [20]. The importance of structural and dynamic separation will be the subject of further studies involving NMR, MD, and neutron scattering.

While qualitative conclusions are obtained from this preliminary study, a better understanding of demixing or microphase separations will include MD simulations of the system surface/radical/adsorbate, as well as more thorough investigations with surfaces containing embedded paramagnetic centers such as rocks, clays, and particularly cements where both the specific surface and metal concentration may be sufficiently high to quantify the individual contributions to relaxation, assuming a well‐defined polarity of the solid surface. These approaches will help in the development of a more generally applicable model for NMR surface relaxation of adsorbates at surfaces of various polarity and interaction strength.

## Author Contributions

**Carlos Mattea:** resources, investigation, conceptualization, project administration, supervision. **Hannah Leptin:** software, formal analysis, methodology, validation, visualization, data curation. **Moritz Schmidt:** methodology, validation, software, formal analysis, visualization, data curation. **Siegfried Stapf:** conceptualization, supervision, writing – original draft, writing – review and editing, project administration.

## Funding

The authors have nothing to report.

## Data Availability

The data that support the findings of this study are available from the corresponding author upon reasonable request.

## References

[1] P. Levitz and J.‐P. Korb , “Slow Dynamics of Embedded Fluid in Mesoscopic Confining Systems as Probed by NMR Relaxometry,” European Physical Journal E: Soft Matter and Biological Physics 12 (2003): 29–33. 10.1140/epje/i2003-10046-2 15007676

[2] J. P. Korb , “Nuclear Magnetic Relaxation of Liquids in Porous Media,” New Journal of Physics 13 (2011): 035016.

[3] J. P. Korb , “Multiscale Nuclear Magnetic Relaxation Dispersion of Complex Liquids in Bulk and Confinement,” Progress in Nuclear Magnetic Resonance Spectroscopy 104 (2018): 12–55. 10.1016/j.pnmrs.2017.11.001 29405980

[4] S. Stapf , R. Kimmich , and R.‐O. Seitter , “Proton and Deuteron Field‐Cycling NMR Relaxometry of Liquids in Porous Glasses: Evidence for Lévy‐Walk Statistics,” Physical Review Letters 75 (1995): 2855–2858. 10.1103/PhysRevLett.75.2855 10059422

[5] S. Stapf , R. Kimmich , R.‐O. Seitter , A. I. Maklakov , and V. D. Skirda , “Proton and Deuteron Field‐Cycling NMR Relaxometry of Liquids Confined in Porous Glasses,” Colloids and Surfaces. A, Physicochemical and Engineering Aspects 115 (1996): 107–114.

[6] S. Stapf , R. Kimmich , and R.‐O. Seitter , “Field‐Cycling NMR Relaxometry of Liquid Confined in Porous Glass: Evidence for Lévy Walks,” Magnetic Resonance Imaging 14 (1996): 841–846. 10.1016/s0730-725x(96)00213-5 8970092

[7] T. Zavada and R. Kimmich , “The Anomalous Adsorbate Dynamics at Surfaces in Porous Media Studied by Nuclear Magnetic Resonance Methods. The Orientational Structure Factor and Lévy Walks,” Journal of Chemical Physics 109 (1998): 6929–6939.

[8] T. Zavada , R. Kimmich , J. Grandjean , and A. Kobelkov , “Field‐Cycling NMR Relaxometry of Water in Synthetic Saponites: Lévy Walks on Finite Planar Surfaces,” Journal of Chemical Physics 110 (1999): 6977–6981.

[9] R. Kimmich , “Strange Kinetics, Porous Media, and NMR,” Chemical Physics 284 (2002): 253–285.

[10] J. Tritt‐Goc , A. Rachocki , and M. Bielejewski , “The Solvent Dynamics at Pore Surfaces in Molecular Gels Studied by Field‐Cycling Magnetic Resonance Relaxometry,” Soft Matter 10 (2014): 7810–7818. 10.1039/c4sm01140f 25145831

[11] M. J. Beira , M. P. Silva , M. Condesso , et al., “Molecular Order and Dynamics of Water in Hybrid Cellulose Acetate‐Silica Asymmetric Membranes,” Molecular Physics 117 (2019): 975–982.

[12] B. Gizatullin , C. Mattea , I. Shikhov , C. Arns , and S. Stapf , “Modelling Molecular Interactions With Wetting and Non‐wetting Rock Surfaces by Combining Electron Paramagnetic Resonance and NMR Relaxometry,” Langmuir 38 (2022): 11033–11053. 10.1021/acs.langmuir.2c01681 36047994

[13] S. Stapf , I. Shikhov , C. Arns , B. Gizatullin , and C. Mattea , “Dipolar NMR Relaxation of Adsorbates on Surfaces of Controlled Wettability,” Magnetic Resonance Letters 3 (2023): 220–231. 10.1016/j.mrl.2023.02.001 PMC12406558 40918634

[14] B. Gizatullin , C. Mattea , and S. Stapf , “Radicals on the Silica Surface: Probes for Studying Dynamics by Means of Fast Field Cycling Relaxometry and Dynamic Nuclear Polarization,” Magnetic Resonance Letters 3 (2023): 256–265. 10.1016/j.mrl.2023.03.006 PMC12406502 40918635

[15] B. Gizatullin , C. Mattea , and S. Stapf , “Effect of Exchange Dynamics on the NMR Relaxation of Water in Porous Silica,” Journal of Physical Chemistry Letters 15 (2024): 11335–11341. 10.1021/acs.jpclett.4c02590 PMC11571216 39499855

[16] B. Gizatullin , C. Mattea , S. Stapf , T. Wissel , and G. Buntkowsky , “NMR Relaxation Dispersion of Liquids Adsorbed on Modified Surfaces of SBA‐15 Mesoporous Silica,” Journal of Physical Chemistry C 128 (2024): 8785–8796.

[17] J. Ward‐Williams and L. F. Gladden , “Insights Into Adsorption Behavior of Binary Liquid Mixtures in Porous Media Using Fast Field‐Cycling NMR,” Magnetic Resonance Imaging 56 (2019): 57–62. 10.1016/j.mri.2018.08.007 30228016

[18] J. Ward‐Williams , L. C. M. Rozing , M. Appel , M. D. Mantle , A. J. Sederman , and L. F. Gladden , “Exploring the Competitive Adsorption of Water−Ethanol Mixtures Within Mesoporous Silicas,” Journal of Physical Chemistry C 129 (2025): 18179–18189.

[19] S. Stapf , N. Siebert , T. Spalek , V. Hartmann , B. Gizatullin , and C. Mattea , “Binary Fluids in Mesoporous Materials: Phase Separation Studied by NMR Relaxation and Diffusion,” Magnetic Resonance Letters 3 (2023): 108–117. 10.1016/j.mrl.2023.03.002 PMC12406583 40917999

[20] N. Siebert , A. Sara , P. Merle , C. Mattea , S. Stapf , and C. Dressler , “Evidence for Demixing of Miscible Binary Liquids in Mesoporous Glass,” Physical Chemistry Chemical Physics *in press*, 10.1039/D6CP00818F. 42334338

[21] L.‐P. Hwang and J. H. Freed , “Dynamic Effects of Pair Correlation Functions on Spin Relaxation by Translational Diffusion in Liquids,” Journal of Chemical Physics 63 (1975): 4017–4025.

[22] M. C. Capel‐Sanchez , L. Barrio , J. M. Campos‐Martin , and J. L. G. Fierro , “Silylation and Surface Properties of Chemically Grafted Hydrophobic Silica,” Journal of Colloid and Interface Science 277 (2004): 146–152. 10.1016/j.jcis.2004.04.055 15276051

[23] K. E. Washburn , “Relaxation Mechanisms and Shales,” Concepts in Magnetic Resonance Part A 43A, no. 3 (2014): 57–78.

[24] K. E. Washburn , E. Anderssen , S. J. Vogt , et al., “Simultaneous Gaussian and Exponential Inversion for Improved Analysis of Shales by NMR Relaxometry,” Journal of Magnetic Resonance 250 (2015): 7–16. 10.1016/j.jmr.2014.10.015 25459882

[25] C. Cadar , A. Cretu , M. Moldovan , C. Mattea , S. Stapf , and I. Ardelean , “NMR T1–T2 Correlation Analysis of Molecular Absorption Inside a Hardened Cement Paste Containing Silanised Silica Fume,” Molecular Physics 117 (2019): 1000–1005.

[26] E. Almagor and G. Belfort , “Relaxation Studies of Adsorbed Water on Porous Glass: I. Varying Coverages and Pore Size at Constant Temperature,” Journal of Colloid and Interface Science 66 (1978): 146–152.

[27] X.‐H. Ren , S. Stapf , H. Kühn , D. E. Demco , and B. Blümich , “Molecular Mobility in Fixed‐Bed Reactors Investigated by Multiscale NMR Techniques,” Magnetic Resonance Imaging 21 (2003): 261–268. 10.1016/s0730-725x(03)00134-6 12850717

[28] S. Stapf , R. Kimmich , and J. Niess , “Microstructure of Porous Media and Field‐Cycling Nuclear Magnetic Resonance Relaxation Spectroscopy,” Journal of Applied Physics 75 (1994): 529–537.

[29] R. Kimmich , S. Stapf , P. T. Callaghan , and A. Coy , “Microstructure of Porous Media Probed by NMR Techniques in Sub‐Micrometer Length Scales,” Magnetic Resonance Imaging 12 (1994): 339–343. 10.1016/0730-725x(94)91552-0 8170335

[30] J. R. Zimmerman and W. E. Brittin , “Nuclear Magnetic Resonance Studies in Multiple Phase Systems: Lifetime of a Water Molecule in an Adsorbing Phase on Silica Gel,” Journal of Physical Chemistry 61 (1957): 1328–1333.

[31] K. R. Brownstein and C. E. Tarr , “Importance of Classical Diffusion in NMR Studies of Water in Biological Cells,” Physical Review A 19 (1979): 2446–2453.

[32] O. V. Bychuk and B. O'Shaughnessy , “Anomalous Surface Diffusion – A Numerical Study,” Journal of Chemical Physics 101 (1994): 772–780.

[33] J. Ward‐Williams , J.‐P. Korb , and L. F. Gladden , “Insights Into Functionality‐Specific Adsorption Dynamics and Stable Reaction Intermediates Using Fast Field Cycling NMR,” Journal of Physical Chemistry C 122 (2018): 20271–20278.

